# Effect of serotonin receptor gene variants on substance use disorders

**DOI:** 10.1080/07853890.2024.2445779

**Published:** 2024-12-28

**Authors:** Laith AL-Eitan, Hana Abu Kharmah, Mansour Alghamdi

**Affiliations:** a Department of Biotechnology and Genetic Engineering, Jordan University of Science and Technology, Irbid, Jordan; b Department of Anatomy, College of Medicine, King Khalid University, Abha, Saudi Arabia; c Genomics and Personalized Medicine Unit, College of Medicine, King Khalid University, Abha, Saudi Arabia

**Keywords:** Polymorphisms, serotonin receptors, substance use, genetics

## Abstract

**Background:**

Substance use disorders are multifaceted conditions influenced by both genetic and environmental factors. Serotonergic pathways are known to be involved in substance use disorder susceptibility, with genetic markers within serotonin receptor genes identified as potential risk factors.

**Methods:**

To further explore this relationship, we conducted a study to investigate the association between several polymorphisms in five serotonin receptor genes (*HTR1B*, *HTR2A/B*, *HTR3A/B*) and substance use disorders (SUD) in Jordanian males by sequencing genotypes in 496 SUD patients and 496 healthy controls.

**Results:**

Our findings revealed an allelic association between rs9567735 in the *HTR2A* gene and rs17586428 in the *HTR2B* gene with SUD. Haplotype analysis also showed that one haplotype of the *HTR2A* gene and four haplotypes of the five included genes were significantly associated with SUD risk. Moreover, we found that motives for substance use were correlated with single nucleotide polymorphisms SNPs rs1923882 and rs1150226, with the latter SNP also being associated with smoking.

**Conclusion:**

These findings suggest that genetic variants of human 5-HT receptor genes may affect individual susceptibility to SUD in Jordan. However, further studies with larger sample sizes and additional variants in the same or different genes must confirm these findings.

## Introduction

Substance use disorders (SUD), also denominate ­addiction when it is severe, imply different forms of dependence on various types of substances, including Cannabinoids, Opioids [1–3], Nicotine, Alcohol, Depressants, Stimulants, and Hallucinogens [4]. SUD are multifactorial neuropsychiatric disorders that are influenced by environmental and genetic factors [5,6]. Personal susceptibility is a crucial factor in SUD development [4,7]. Starting from the presence of psychiatric disorders, which carry a significantly increased risk of developing addiction (dual disorder) [5,8] to genetic factors that influence the combination of aspects, such as the difference in comorbidities between different substances [6]. Dual diagnosis, or dual disorders, are terms used to define the presence of SUD and another psychiatric disorder in an individual simultaneously [7]. These disorders impact individuals and their families throughout their lifespan, regardless of race, gender, age, culture, ethnicity, spiritual belief, or socioeconomic status. The high incidence has been documented among people with a dual diagnosis. The National Survey on Drug Use and Health reveals that approximately 50% of SUD individuals also have coexisting psychiatric disorders [8]. These patients with a dual diagnosis have more severe and persistent symptoms [7], are more often homeless [9], and experience treatment-resistant [9–14].

Risk factors for SUD include being male, having a family history of SUD, young age, and having a comorbid psychiatric disorder such as major depression, bipolar disorder (BPD), and post-traumatic stress disorder [15]. Regarding genetic vulnerability to SUD, the serotonergic system could explain how genetics can influence substance use [16]. Although the dopaminergic system is the prevalent pathway for elucidating the etiology and onset of SUD, the serotonergic system plays a crucial role in SUD development and progression [17]. Serotonin maintains synaptic flexibility. In addition to its role in learning and memory, it is also essential in motivational and support processes [18]. The central serotonergic system controls mood and is involved in several neuropsychiatric behaviors [19]. Altered serotonin function was observed after cocaine drawing, and decreased sensitivity of serotonin receptors was reported in heroin addicts even after drug cessation [20].

One of the interesting human receptors is the 5-HT (5-hydroxytryptamine), a G protein-coupled serotonin receptor, which is interesting in investigating SUD [21,22]. They are involved in increased alcohol and drug consumption. In addition, 5-HT is implicated in psychological behaviors such as suicide and aggression [23]. However, clinical variations are affected by an individual’s genetic background, which explains the variation in the results of many studies among different populations [24]. This diversity reveals the adequacy of pharmacogenomics for modulating reward pathways by tampering with serotonergic signaling. Some selective serotonin reuptake inhibitor (SSRI) classes, such as fluoxetine, have been reported to reduce the self-administration of cocaine. Moreover, 5-HT (precursors, uptake inhibitors, and intracerebral and postsynaptic 5-HT agonists) linked with alcohol decreased intake [25,26].

This study focused on five 5-HT receptors (*HTR1B*, *HTR2A*, *HTR2B*, *HTR3A*, and *HTR3B*). The human *HTR1B* (*5‐HT1B*) gene on chromosome 6 encodes for the 5‐hydroxytryptamine (serotonin) receptor 1 B, a presynaptic autoreceptor that mediates serotonin production and discharge regulation. *HTR1B* polymorphisms in coding and regulatory regions have been implicated in SUD risks such as alcohol and drug abuse [27,28]. *HTR1B* is a gene of interest that can act as a putative regulator of different psychiatric disorders [29]. On the other hand, *HT1B* stimulation enhances the cocaine reward pathways by reducing 5HT release. In this regard, the absence of the 5 *HT1B* receptor was correlated with increased self-administration of cocaine and increased alcohol intake [30].

5*-*HT2A is another 5-HT serotonin receptor from the G protein superfamily identified as a good candidate factor for the etiology of SUD. This gene contains 62 kb pairs on chromosome 13 [31,32]. The presynaptic and postsynaptic schemes of *HTR2A* suggest the involvement of the receptor in the reward system [33,34]. The vital role of *5-HT2A* is attributed to its features as an action site for specific drugs. The activation of *HTR2A* can decrease alcohol consumption. Single nucleotide variants (rs6313 and rs6311) are the most commonly studied single nucleotide variants, and a plausible association between alcohol and heroin dependence has been found [31]. *HTR2A* is also involved in nicotine dependence, as nicotine enhances and elevates the released serotonin. Due to functional genetic polymorphisms in its receptors, the altered serotonergic system may impact smoking dependence, such as the *HTR2A* A-1438G single nucleotide polymorphism SNP. The A allele was found to promote the transcriptional activity of *HTR2A* [32,35]. However, HTR2B on chromosome 2 needs to be adequately investigated. However, a significant mutation in the gene stop codon (*HTR2B* Q20*) was found to be associated with impulsive behavior and cognitive impulsivity due to depletion in receptor expression [36].

*HTR3A* and *HTR3B* genes span 90-Kb on chromosome 11 and are expressed in loci involved in SUD [20]. HT3 receptors have been implicated in alcohol dependence; it has been reported that alcoholism manipulates gene expression and stimulates behavioral changes [37].

The purpose of this study was to clarify the possible association between several single nucleotide polymorphisms (SNPs) within serotonin receptor genes (*HTR1B*, *HTR2A/B*, *HTR3A/B*) and SUD in Jordanian males.

## Material and methods

### Subject and genotyping

A total of 496 males who were diagnosed with different forms of substance use disorders (SUD) and 496 unrelated healthy males were recruited for the current study. The case group was nominated according to the substance use criteria of the Manual of Mental Disorders (DSM-IV) (APA, 2013) [38]. All subjects were Jordanians of Arab origin; the case cohort was obtained from the Jordanian Ministry of Health rehabilitation centers. This study is in accordance with the Public Security Directorate (J/2/46/21546), the Jordanian Ministry of Health (M/R/180057), the Institutional Review Board of Jordan University of Science and Technology (43/114/2018), the King Abdullah University Hospital (43/114/2018), and the Declaration of Helsinki. All participants provided written informed consent for participation in the study and the publication of the results. Much medical and demographic data was collected from the patient’s medical records.

For the genotyping method, DNA was extracted according to the Wizard^®^ Genomic DNA Purification Kit using peripheral blood samples. PCR and sequencing techniques were accomplished abroad by the Australian Genome Research Facility (AGRF) using the Agena Bioscience MassARRAY^®^ on a Compact Spectrometer, iPLEX GOLD chemistry. Out of Jordan’s total ­population of 9,531,712, approximately 134,947 adult males have SUD, with a prevalence of 2.5% (https://www.who.int/publications/m/item/jordan-who-special-initiative-for-mental-health). OpenEpi software was used to calculate the required sample size with a 95% confidence interval. A prevalence of substance use disorder of 2.5%, a design effect of 1%, and a precision of 3% were considered when calculating the sample size, which was 105 participants. The study sample size exceeded the required number of 105 participants, as 496 individuals were included in the cases.

### Statistical analysis

SNPStats online software was used to investigate various genetic models, haplotype frequency estimates, and Hardy-Weinberg equilibrium HWE (https://www.snpstats.net/start.htm). The minor allele frequencies were also investigated using SNPStats. The Statistical Package for the Social Sciences (SPSS) (version 25.0; SPSS, Inc., Chicago, IL, USA) was used to detect the relationship between different substance use disorder features and the investigated variants. Statistical significance was set at *p* < 0.05.

The multiple testing correction using the Bonferroni correction, where the significance threshold was set at α/n, where α equals 0.05 and n represents the number of tests, and to sustain the overall *P*-value at a significance level of 0.005 or less [39].

## Results

### Subject description

The study cohort was previously analyzed for different genetic markers [3,40,41]. All subjects included in this study were unrelated males from Jordan. Healthy individuals had no history of substance use or psychiatric disorders, with a mean age (± Sd.) of 29 ± 6.9. The patient group was hospitalized in 2018 for eight months in rehabilitation centers in Jordan. Table 1 summarizes the essential clinical features of the patients.

**Table 1.** Cases characteristics.

**Mean Age (year)**	28.6 ± 9.1
**Mean Age at onset (year)**	24.3 ± 8.9
**mean duration of substance use (month)**	5.6 ± 5.1
**Smoking**	88.2%
**Type of the used substance**	
synthetic cannabinoids	48.0%
Cannabinoids	20.0%
Amphetamine	5.8%
Alcohol	5.3%
benzodiazepines	4.3%
Opiates	4.5%
Cocaine	1.1%
Mixed substances (two or more)	11.3%
**Route of substance use**	
Smoking	70.9%
oral	15.2%
Injection	4.3%
**Motive for SUD**	
Friends	45.6%
curiosity	30.1%
circumstances	23.2%
For joy	0.6%
Prescribed by doctors	0.4%
**Number of used substances**	
Alcohol alone	5.5%
One	83.2%
Two or more	11.3%

### Polymorphism’s characteristics

This study included 11 genetic variants within five serotonin receptor genes (*HTR1B*, *HTR2A/B*, *HTR3A/B*), as shown in Table 2. Data regarding the polymorphisms are also included in the corresponding table: chromosomal positions, minor alleles, their frequencies, and the consequences, in addition to the *P*-value of HWE for single nucleotide polymorphisms (SNPs). One SNP (rs1176713) of the *HTR3B* gene was excluded from the study as it failed to accomplish the HWE (*P*-value < 0.05).

**Table 2.** Minor allele frequencies among cases and healthy controls and the HWEc *P*-value of the candidate gene polymorphisms.

Gene	SNP ID	SNV position^a^	Consequence	Cases (*n* = 496)			Controls (*n* = 496)	
				MA^b^	MAF^c^	HWE^d^*P*-value	MAF^c^	HWE^d^*P*-value
**HTR1B**	rs6296	6:77462543	Synonymous variant	G	0.29	0.59	0.28	0.10
**HTR2A**	rs1923882	13:46837526	Intron variant	T	0.21	0.79	0.21	0.59
	rs6561333	13:46846177	Intron variant	T	0.31	0.05	0.33	0.55
	rs9567735	13:46845069	Intron variant	G	0.21	0.11	0.17	0.26
**HTR2B**	rs17586428	2:231124141	Intron variant	G	0.15	1.0	0.11	0.82
	rs6437000	2:231112813	Intron variant	A	0.44	0.23	0.45	0.53
**HTR3A**	rs1150226	2:231112813	Intron variant	A	0.10	0.81	0.11	1.0
	rs897687	11:113985089	Intron variant	G	0.05	0.28	0.05	0.65
	rs1176713	11:113989703	Synonymous variant	G	0.17	0.88	0.21	0.013
**HTR3B**	rs11606194	11:113910259	Intron variant	C	0.04	1.0	0.03	1.0
	rs1176744	11:113932306	Missense variant	C	0.39	0.57	0.36	0.05

^a^Chromosome positions are based on NCBI human genome assembly build.

^b^MA: minor allele.

^c^MAF: minor allele frequency.

^d^HWE: Hardy—Weinberg equilibrium.

Serotonin receptor genes are good candidates for studying the etiology of psychiatric disorders by investigating the genetic markers within these genes. The SNPs in the current study were preferred because of their underlying effect on the receptors’ biological functions in manipulating the serotonergic system and their critical locations on the genes. In addition, according to the literature, there is persuasive evidence regarding the implication of serotonin receptors on SUD risk.

### Genetic association and haplotype analyses

Table 3 shows the distribution of the alleles and genotypes of the serotonin receptor genes among the cases and controls. A statistically significant association was observed between the alleles of rs9567735 in *HTR2A* and SUD (*P*-value = 0.01). However, genotypic analyses for this variant revealed no correlation with SUD (*P*-value = 0.06). The variant allele (G) was distributed more among cases (0.21) than among controls (17%). This finding suggests a possible implication of rs9567735 in the increased risk of SUD. Another allelic association with SUD was detected in this study: rs17586428 of *HTR2B* (*P*-value = 0.01). Statistics revealed that the G allele of this variant may confer an increased risk of SUD development and progression. The SNPs of *HTR1B*, *HTR3A,* and *HTR3B* were not correlated with SUD in this study (Table 3). After applying the Bonferroni correction with a *P*-value threshold of 0.005, no statistically significant genetic associations were observed between the SNPs and SUD.

**Table 3.** Genetic associations between the polymorphisms and SUD.

Gene	SNP ID	Allelic and genotypic frequencies in cases and controls				
		Allele/genotype	Cases (*n* = 495)	Controls (*n* = 497)	OR (CI: 95%)	*P*-value*
**HTR1B**	rs6296	C	689 (0.71)	713 (0.72)		0.07
		G	285 (0.29)	271 (0.28)		
		CC	241 (0.49)	266 (0.54)	1.00	0.18
		CG	207 (0.43)	181 (0.37)	1.26 (0.97-1.65)	
		GG	39 (0.08)	45 (0.09)	0.96 (0.60-1.52)	
**HTR2A**	rs1923882	C	778 (0.79)	778 (0.79)		1.0
		T	210 (0.21)	210 (0.21)		
		CC	305 (0.62)	304 (0.62)	1.00	0.98
		CT	168 (0.34)	170 (0.34)	0.98 (0.76-1.29)	
		TT	21 (0.04)	20 (0.04)	1.05 (0.56-1.97)	
	rs6561333	C	669 (0.69)	658 (0.67)		0.35
		T	307 (0.31)	330 (0.33)		
		CC	239 (49%)	222 (44.9%)	1.00	0.39
		CT	191 (39.1%)	214 (43.3%)	0.83 (0.63-1.08)	
		TT	58 (11.9%)	58 (11.7%)	0.93 (0.62-1.40)	
	rs9567735	A	763 (0.79)	815 (0.83)		0.01
		G	207 (0.21)	167 (0.17)		
		AA	306 (63.1%)	342 (69.7%)	1.00	0.06
		AG	151 (31.1%)	131 (26.7%)	1.29 (0.97-1.71)	
		GG	28 (5.8%)	18 (3.7%)	1.74 (0.94-3.21)	
**HTR2B**	rs17586428	A	845 (0.85)	881 (0.89)		0.01
		G	145 (0.15)	109 (0.11)		
		A/A	360 (72.7%)	391 (79%)	1.00	0.05
		G/A	125 (25.2%)	99 (20%)	1.37 (1.02-1.85)	
		G/G	10 (2%)	5 (1%)	2.17 (0.74-6.42)	
	rs6437000	C	544 (56%)	537 (55%)		0.63
		A	434 (44%)	447 (45%)		
		A/A	158 (32.3%)	150 (30.5%)	1.00	0.82
		C/A	228 (46.6%)	237 (48.2%)	0.91 (0.68-1.22)	
		C/C	103 (21.1%)	105 (21.3%)	0.93 (0.66-1.32)	
**HTR3A**	rs1150226	G	887 (90%)	886 (89%)		0.83
		A	101 (10%)	104 (11%)		
		G/G	397 (80.4%)	396 (80%)	1.00	0.94
		G/A	93 (18.8%)	94 (19%)	0.99 (0.72-1.36)	
		A/A	4 (0.8%)	5 (1%)	0.80 (0.21-2.99)	
	rs897687	A	942 (95%)	934 (95%)		0.41
		G	46 (0.05%)	54 (0.05%)		
		A/A	450 (91.1%)	442 (89.5%)	1.00	0.68
		G/A	42 (8.5%)	50 (10.1%)	0.83 (0.54-1.27)	
		G/G	2 (0.4%)	2 (0.4%)	0.98 (0.14-7.00)	
**HTR3B**	rs11606194	T	952 (0.96)	943 (0.97)		0.11
		C	38 (0.04)	25 (0.03)		
		TT	457 (92.3%)	459 (94.8%)	1.00	0.11
		TC	38 (7.7%)	25 (5.2%)	1.53 (0.91-2.57)	
	rs1176744	A	586 (0.61)	628 (0.64)		0.12
		C	378 (0.39)	350 (0.36)		
		A/A	181 (37.5%)	212 (43.4%)	1.00	0.18
		C/A	224 (46.5%)	204 (41.7%)	1.29 (0.98-1.69)	
		C/C	77 (16%)	73 (14.9%)	1.24 (0.85-1.80)	

*P-value <0.05 considered as significant.

OR: odd ratio.

Further genetic association model analyses were also performed (Table 4). Only the Dominant model of rs17586428 in *HTR2B* A/A vs. G/A-G/G was linked with SUD risk (*P*-value =0.02). However, according to the Bonferroni correction, no significant association was observed. Table 5 presents haplotype analyses of the SNPs investigated in this study. The CCG of the *HTR2A* block (rs1923882, rs6561333, and rs9567735) was slightly associated with SUD risk (*P*-value= 0.047). The haplotype frequency within cases was higher than that among controls, suggesting that the haplotype above is an increased risk factor for SUD.

**Table 4.** Different genetic model analysis between the investigated SNPs and SUD.

Gene	SNP ID	Model	Genotype	Cases (%)	Controls (%)	OR (95% CI)	*P*-value
HTR1B	rs6296	Dominant	C/C C/G-G/G	241 (49.5%) 246 (50.5%)	266 (54.1%) 226 (45.9%)	1.00 1.20 (0.93–1.54)	0.15
		Recessive	C/C-C/G G/G	448 (92%) 39 (8%)	447 (90.8%) 45 (9.2%)	1.00 0.86 (0.55–1.35)	0.52
HTR2A	rs1923882	Dominant	C/C C/T-T/T	305 (61.7%) 189 (38.3%)	304 (61.5%) 190 (38.5%)	1.00 0.99 (0.77–1.28)	0.95
		Recessive	C/C-C/T T/T	473 (95.8%) 21 (4.2%)	474 (96%) 20 (4%)	1.00 1.05 (0.56–1.97)	0.87
	rs6561333	Dominant	C/C C/T-T/T	239 (49%) 249 (51%)	222 (44.9%) 272 (55.1%)	1.00 0.85 (0.66–1.09)	0.20
		Recessive	C/C-C/T T/T	430 (88.1%) 58 (11.9%)	436 (88.3%) 58 (11.7%)	1.00 1.01 (0.69–1.49)	0.94
	rs9567735	Dominant	A/A G/A-G/G	306 (63.1%) 179 (36.9%)	342 (69.7%) 149 (30.4%)	1.00 1.34 (1.03–1.75)	0.03
		Recessive	A/A-G/A G/G	457 (94.2%) 28 (5.8%)	473 (96.3%) 18 (3.7%)	1.00 1.61 (0.88–2.95)	0.12
HTR2B	rs17586428	Dominant	A/A G/A-G/G	360 (72.7%) 135 (27.3%)	396 (80%) 99 (20%)	1.00 1.41 (1.05–1.89)	0.02
		Recessive	A/A-G/A G/G	485 (98%) 10 (2%)	490 (99%) 5 (1%)	1.00 2.02 (0.69–5.96)	0.19
	rs6437000	Dominant	C/C C/A-A/A	158 (32.3%) 331 (67.7%)	150 (30.5%) 342 (69.5%)	1.00 0.92 (0.70–1.20)	0.54
		Recessive	C/C-C/A A/A	386 (78.9%) 103 (21.1%)	387 (78.7%) 105 (21.3%)	1.00 0.98 (0.72–1.34)	0.92
HTR3A	rs1150226	Dominant	G/G G/A-A/A	397 (80.4%) 97 (19.6%)	396 (80%) 99 (20%)	1.00 0.98 (0.71–1.34)	0.89
		Recessive	G/G-G/A A/A	490 (99.2%) 4 (0.8%)	490 (99%) 5 (1%)	1.00 0.80 (0.21–3.00)	0.74
	rs897687	Dominant	A/A G/A-G/G	450 (91.1%) 44 (8.9%)	442 (89.5%) 52 (10.5%)	1.00 0.83 (0.54–1.27)	0.39
		Recessive	A/A-G/A G/G	492 (99.6%) 2 (0.4%)	492 (99.6%) 2 (0.4%)	1.00 1.00 (0.14–7.13)	N/A
HTR3B	rs1176744	Dominant	A/A C/A-C/C	181 (37.5%) 301 (62.5%)	212 (43.4%) 277 (56.6%)	1.00 1.27 (0.98–1.65)	0.07
		Recessive	A/A-C/A C/C	405 (84%) 77 (16%)	416 (85.1%) 73 (14.9%)	1.00 1.08 (0.76–1.53)	0.65

*P*- Value <0.025 was considered as significant after performing Bonferroni correction.

OD: odd ratio; CI: Confidence interval.

N/A: Not applicable.

**Table 5.** Haplotype association with SUD.

HTR2A Block (rs1923882, rs6561333, rs9567735)	Frequency of block	Frequency ratio % (case :control)	Odds ratio (95%) CI	*P*-value
**CCA**	0.327	0.3164: 0.3381	1.00	N/A
**CTA**	0.270	0.2611: 0.2794	1.01 (0.79–1.28)	0.96
**CCG**	0.189	0.2098: 0.17	1.30 (1.00–1.67)	0.047
**TCA**	0.156	0.156: 0.1577	1.05 (0.78–1.42)	0.73
**TTA**	0.054	0.0535: 0.0549	1.04 (0.65–1.64)	0.88
**CTG** *	0.002	0.0031: 0	65418018.90 (65418018.87–65418018.92)	<0.0001
**HTR2B block (rs17586428, rs6437000)**				
**AC**	0.5513	0.5576: 0.5449	1.00	N/A
**AA**	0.3204	0.2959: 0.345	0.84 (0.69–1.02)	0.072
**GA**	0.1283	0.1465: 0.1101	1.32 (1.00–1.73)	0.052
**HTR3A block (rs1150226, rs897687)**				
**GA**	0.8889	0.8893: 0.8885	1.00	N/A
**AA**	0.0604	0.0643: 0.0565	1.14 (0.78–1.66)	0.50
**AG**	0.0432	0.0378: 0.0486	0.78 (0.51–1.21)	0.28
**GG** *	0.0075	0.0086: 0.0064	1.28 (0.47–3.50)	0.62
**HTR3B Block (rs11606194, rs1176744)**				
**TA**	0.6139	0.5972: 0.6305	1.00	N/A
**TC**	0.354	0.3644: 0.3436	1.10 (0.91–1.32)	0.33
**CC**	0.0234	0.0314: 0.0154	2.11 (1.07–4.18)	0.03
CA*	0.0087	0.007: 0.0104	0.73 (0.22–2.43)	0.61
				
**Haplotype (rs6296, rs1923882,rs6561333,rs9567735, rs17586428,rs6437000, rs1150226, rs897687, rs11606194, rs1176744)**				
**CCCAAAGATA**	0.0497	0.018: 0.0721	0.14 (0.05–0.39)	2e-04
**CCCGACGATA**	0.0432	0.0318: 0.0588	0.37 (0.17–0.84)	0.017
**CTTAACGATA**	0.0174	0.0076: 0.0183	0.15 (0.04–0.62)	0.009
**GTCAACGATC**	0.0168	0.0066: 0.013	0.16 (0.04–0.66)	0.011

^*^ Rare haplotype.

N/A: Not applicable.

Moreover, the *HTR3B* Block (rs11606194, rs1176744) created one significant haplotype (CC) with a *P*-value of 0.03. These results suggest that this haplotype within individuals is likely to influence the risk of SUD. Four substantial haplotypes were generated from all SNPs included in this study. A plausible relationship between these haplotypes and SUD was also identified (Table 5). All four haplotypes were distributed within the cases lower than those among the controls and may serve as protective factors against SUD risk. Only one haplotype from all SNPs, with a *P*-value of 2e-04, was considered statistically significant after applying the Bonferroni correction.

This study investigated the influence of genetic markers on SUD risk in the presence of several features. Table 6 illustrates the relationship between SUD features and serotonin receptor gene variants. Motives for substance use were correlated with SNPs rs1923882 (*P*-Value = 0.004) and rs1150226 (*P*-Value = 0.037). The latter SNP was also associated with smoking (*P*-Value = 0.001). In addition, SNP rs6296 was associated with the route of substance use (*P*-Value= 0.048). Moreover, only the associations of rs1923882 with motives for substance use and rs1150226 with smoking were considered significant after applying the Bonferroni correction.

**Table 6.** Association between several SUD features and serotonin receptors genetic variants.

HTR1B polymorphisms	*SUD features*							
	Age of cases*	Age at onset*	Duration of substance use (years)*	Motives for substance use**	Types of substances**	Smoking**	Route of substance use**	Number of substances**
rs6296	0.432	0.218	0.561	0.266	0.507	0.152	**0.048**	0.181
**HTR2A polymorphisms**								
rs1923882	0.868	0.967	0.872	0.004	0.765	0.319	0.468	0.267
rs6561333	0.525	0.331	0.510	0.716	0.375	0.709	0.445	0.578
rs9567735	0.932	0.986	0.302	0.368	0.681	0.691	0.767	0.269
**HTR2B polymorphisms**								
rs17586428	0.674	0.658	0.597	0.775	0.683	0.480	0.523	0.666
rs6437000	0.165	0.227	0.971	0.221	0.519	0.726	0.880	0.606
**HTR3A polymorphisms**								
rs1150226	0.257	0.323	0.895	0.037	0.782	0.001	0.422	0.341
rs897687	0.721	0.946	0.895	0.895	0.654	0.112	0.261	0.081
**HTR3B polymorphism**								
rs11606194	0.410	0.243	0.454	0.657	0.614	0.812	0.941	0.894
rs1176744	0.214	0.567	0.755	0.200	0.315	0.644	0.438	0.294

*Analysis of variance (ANOVA) was used to determine the association.

**Pearson’s chi-squared test was used to determine the association.

## Discussion

SUD comprises many chronic relapsing illnesses that reflect tolerance and physical dependence on abused substances [40]. SUD present clinically comorbidly with other psychiatric disorders [42–44] and often further complicate the course of these diseases. Moreover, SUD also have a wide range of effects on many organ systems beyond the brain.

The involvement of serotonin receptors in SUD has been indicated in previous genetic studies. The 5HTR subtypes have been implicated in substance dependence and relative behavior. However, inconsistent findings have been obtained, which is reasonable because of the genetic background differences between various ethnic groups [31]. Common genetic variants within the *5HTR* genes play pivotal roles in the development of SUD. In this study, five *5HTR* genes encoding serotonin receptors (*HTR1B*, *HTR2A*/B, *HTR3A/B*) were studied regarding the risk of SUD in Jordanian males.

Our genetic analysis revealed that the SNP rs6296 of *HTR1B* (G861C) was not implicated in SUD. consistent with another study conducted on a Japanese population that investigated three polymorphisms of *HTR1B* and found no correlation between these variants and Methamphetamine Dependence [25]. Another study demonstrated that rs6296 lacks an association with cocaine or alcohol abuse or dependence in the US [45]. Otherwise, in Finnish and southwestern American Indian populations, rs6296 was concomitant with antisocial alcoholism [46]. The large effect of some genetic variants on a specific population may be the cause of differences in association between different populations. Xia et al. examined 14 SNPs in *HTR1B* in the northern Han Chinese population and found that *HTR1B* polymorphisms are associated with schizophrenia [40]. In the same population, another study found that the G861C polymorphism is associated with heroin dependence [47]. This may be due to the comorbidity of SUD with other psychiatric disorders.

A meta-analysis and systematic review found that SUD is linked to higher levels of oxidant markers and lower antioxidant markers compared to healthy controls. [48]. It is generally accepted that oxidative stress is a common factor involved in the pathogenesis and pathophysiology of psychiatric disorders [49,50]. Furthermore, the antioxidant levels appear to correlate with the severity of psychiatric disorders [51], and treatment with antioxidants has been proven to enhance psychiatric symptoms in certain clinical cases. [52,53]. Serotonin, an important neurotransmitter, is also known to contribute to the etiology of psychiatric diseases and several neurodegenerative diseases [54]. A study in African American/Caucasian versus Hispanic/Asian/other populations found a significant association of the *HTR1B* G861C gene locus with substance use disorders and major depressive disorder. On the other hand, there is no relationship between G861C and alcoholism, bipolar disorder, schizophrenia, or a history of a suicide attempt. The disparate findings may reflect differences between populations, including comorbid psychopathology rates.

Prior studies have addressed the impact of *HTR2A/B* on SUD risk. In addition to its chief role in the psychological system, it also contributes to the pharmacology of psychotic medications [31]. Both rs9567735 and rs17586428 in *HTR2A* and *HTR2B* were associated with SUD in the current study. This analysis suggested that these polymorphisms could increase the risk of SUD development and progression [55]. Montalvo-Ortiz et al. revealed an association of *HTR2B* rs17440378 with aggression and impulsivity as a result of cannabis abuse. However, in agreement with our findings, *HTR2B* can be assigned as a significant locus for SUD risk and its induced behaviors [56].

A previous study revealed that *HTR2A* variation influenced both suicide attempts and mood disorders such as bipolar disorder and major depression in the French-speaking population of Quebec [57]. In suicide attempts, the *HTR2A* rs6561333 variant is involved in interactions with a history of sexual and physical abuse. This study failed to find a correlation between the variants of the *HTR3A/B* genes and SUD. In contrast, several studies have reported a compelling association between functional SNPs in genes and SUD [58,59]. The intronic SNP rs11606194 of *HTR3B* is related to heroin addiction in European Americans. Two polymorphisms (HTR1B rs6296 and HTR3A rs1176713) showed genotype association with opioid dependence OD ± Cocaine dependence CD and/or Cocaine dependence CD. The SNP (HTR3A rs897687) showed an association with both OD ± CD and CD. Also, HTR3A rs1150226, was indicated in the combined OD ± CD + CD but was not identified in at least one of the independent analyses. Whereas, rs1923882, rs6561333, and rs9567735 of *HTR2A*, rs17586428, and rs6437000 of HTR2B are not associated with heroin and cocaine addiction [20].

Moreover, *HTR3B* rs1176744 is also associated with alcohol and nicotine dependence [60]. Various SNPs have previously been linked with related phenotypes, including *HTR1B* rs6296 with substance abuse disorder and behavioral problems. Additionally, there is an association between depression and the SNP rs6296 of *HTR1B* [23,61,62], *HTR2A* rs6561333 with cocaine dependence [63], *HTR3B* rs11606194 over time to relapse after smoking cessation and with nicotine dependence [60,63,64], and *HTR3B* rs1176744 with alcohol and nicotine dependence [58–60].

A prior study found a significant association between *HTR3A* and BPD but did not yield any evidence of an association between *HTR3B* and BPD after analysis of a small Chinese sample [65]. Previous data have demonstrated the importance of the serotonergic system for emotional regulation and shown that methylation aberrations of the receptor genes (5HTR1B, 5HTR2A, and 5HTR3A) and the serotonin transporter (SLC6A4) are linked to antisocial traits and BPD [66–69].

The lead finding in this study was a haplotype association with SUD. Significant haplotypes protruded from the investigated SNPs of *HTR2A* and *HTR3B*, and it was anticipated that SUD would increase the risk. Substantial haplotypes emerged from all the SNPs in this study and were suggested to act as protective factors against SUD. However, several SNPs of *5HTR* genes were in linkage disequilibrium (LD) in previous studies, such as rs6296 of *HTR1B* in LD with rs13212041 and *HTR3B* rs1176744 (Tyr129Ser), and *HTR3A* rs1176713, in addition to *HTR3A* rs897687 and *HTR3B* rs11606194 [20]. A correlation between SUD features and the investigated polymorphisms was detected. Motives for substance use were correlated with the SNPs rs1923882 and rs1150226. rs1150226 was also associated with smoking. We propose that GG may increase the risk of SUD development among smokers.

A previous study examined the role of genetic polymorphisms in oxidative stress in Multiple Chemical Sensitivity (MCS), revealing that the AG genotype of paraoxonase 1 PON1 rs662 and the TT and CT genotypes of PON1 rs705379 are involved in anxiety and depression in MCS [70].

Priority is given to unauthorized prescriptions for psychiatric medications [71–73]. Among these medications are SSRIs, which are commonly prescribed because of their many uses [74]. SSRIs, which selectively block the serotonin transporter (5-hydroxytryptamine-5-HT), are used to treat anxiety, post-traumatic stress disorder (PTSD), panic disorder, major depression, bulimia nervosa, and obsessive-compulsive disorder (OCD) [75].

A study conducted by Chiappini et al. aimed to identify misuse, abuse, pharmacovigilance misuse, abuse, dependence, and withdrawal signals related to SSRIs. Using datasets from the FDA Adverse Events Reporting System and EudraVigilance, the study found an increase in annual report trends and similar indications concerning abuse and dependence. SSRIs, despite being non-addictive, can cause withdrawal symptoms long after discontinuation, highlighting the need for clinicians to be aware of potential dependence and withdrawal risks associated with SSRIs [76].

The study reveals those genetic variants in human 5-HT receptor genes may increase the risk of substance use disorder (SUD) development and progression in Jordan. The comprehension of genetic variations could significantly help clinicians in the creation of more effective treatment strategies. Patients with specific polymorphisms, for instance, might benefit more from specific kinds of therapies than other patients. Moreover, serotonin pathways might potentially be affected, given the association between the serotonin receptor gene and SUD. Genetic variations may impact the effectiveness of serotoninergic treatments like SSRIs. Therefore, genetic screening is critical when prescribing medications to modulate serotonin, as patients with specific genetic profiles may respond differently to these treatments. These results highlight the necessity for personalized treatment plans in clinical psychiatry that take the patient’s genetic profile into consideration [67].

To our knowledge, there are no studies in the literature that suggest a direct association between oxidative stress and our gene polymorphisms. Further investigation in this area is required. The study has other limitations that need to be acknowledged. The study’s data may not accurately represent the entire community of drug users in Jordan. The study primarily targeted male participants due to the gender gap, as all of the patients who are registered at the rehabilitation centers are men. Furthermore, females may not seek treatment due to cultural sensitivities and stigma associated with substance use disorders, which could account for the low rate and dearth of female participation. However, we recognize that further research including female participants will be needed to provide a more comprehensive understanding of risk factors for SUD across genders.

Additionally, the study missed assessing psychiatric comorbidity in SUD patients. We were unable to collect data on dual diagnoses in addicted patients. Future studies should focus on this area for a more comprehensive understanding of SUD. It is crucial to comprehend the genetics and epigenetics of addiction processes. Gene variations linked to susceptibility to addiction or resilience to it may be useful in classifying individuals to optimize the effects of prospective treatment or preventative interventions. Moreover, understanding molecular genetic changes resulting from chronic drug exposure or recovery can significantly improve our comprehension of addiction’s neurobiological mechanisms and guide the development of future therapeutic strategies [77]. The integration of genetic findings into the pharmacological treatment of SUD and dual diagnosis holds significant promise. By leveraging knowledge of serotonin gene polymorphisms and oxidative stress, healthcare providers can develop more effective, personalized treatment strategies, potentially improving outcomes for patients with these challenging conditions.

GWAS is a largely successful approach in candidate gene studies. However, the effectiveness of GWAS in diverse populations is a critical question in understanding the relationship between genotypes and SUD. Therefore, further studies and a much larger sample size from both sexes, including patients from different ethnic groups, are needed to validate our findings.

## Conclusion

In summary, this study demonstrated that genetic variants in the human *5-HT* receptor genes may be implicated in individual susceptibility to substance use disorders by manipulating the function or expression of the genes or even being in LD with functional SNPs significant for gene expression or its products.

## Data Availability

The data that support the findings of this study are available from the corresponding author, L.A., upon reasonable request.

## Abbreviations

5-HT: 5-Hydroxytryptamine
AGRF: The Australian Genome Research Facility
CD: Cocaine Dependence
DSM-5: The Fifth Diagnostic and Statistical Manual of Mental Disorders
HWE: Hardy-Weinberg equilibrium
IRP: Institutional Review Board
LD: Linkage Disequilibrium
MCS: Multiple Chemical Sensitivity
OCD: Obsessive-Compulsive Disorder
OD: Opioid Dependence
PCR: Polymerase Chain Reaction
PON1: Paraoxonase 1
PTSD: Post-Traumatic Stress Disorder
SNP: Single Nucleotide Polymorphisms
SPSS: Statistical Package for the Social Sciences
SSRI: Selective Serotonin Reuptake Inhibitors
SUD: Substance Use Disorders
